# Nomogram for prognosis of elderly patients with cervical cancer who receive combined radiotherapy

**DOI:** 10.1038/s41598-023-39764-5

**Published:** 2023-08-16

**Authors:** Wenjuan Chen, Xiaoyi Xia, Xingyun Xie, Yuting Wei, Rongrong Wu, Wenjie Cai, Jinsheng Hong

**Affiliations:** 1 Department of Radiation Oncology, Department of Gynecology, Clinical Oncology School of Fujian Medical University, Fujian Cancer Hospital, Fuzhou, 350014 China; 2 Department of Radiation Oncology, First Hospital of Quanzhou Affiliated to Fujian Medical University, Quanzhou, 362000 China; 3 Department of Radiotherapy, Cancer Center, The First Affiliated Hospital of Fujian Medical University, Fuzhou, 350005 China; 4 Key Laboratory of Radiation Biology of Fujian Higher Education Institutions, The First Affiliated Hospital, Fujian Medical University, Fuzhou, 350005 China; 5 Department of Radiotherapy, National Regional Medical Center, Binhai Campus of the First Affiliated Hospital, Fujian Medical University, Fuzhou, 350212 China

**Keywords:** Cancer, Medical research

## Abstract

This retrospective study identified prognostic factors to help guide the clinical treatment of elderly patients (≥ 65 years) with cervical cancer who had undergone radiotherapy. A personalized model to predict 3- and 5-years survival was developed. A review was conducted of 367 elderly women with cervical cancer (staged II–III) who had undergone radiotherapy in our hospital between January 2012 and December 2016. The Cox proportional hazards regression model was used for survival analysis that considered age, hemoglobin, squamous cell carcinoma antigen, pathologic type, stage, pelvic lymph node metastasis status, and others. A nomogram was constructed to predict the survival rates. The median follow-up time was 71 months (4–118 months). The 3- (5-) years overall, progression-free, local recurrence-free, and distant metastasis-free survival rates were, respectively, 91.0% (84.4%), 92.3% (85.9%), 99.18% (99.01%), and 99.18% (97.82%). The following were significant independent prognostic factors for overall survival: tumor size, pre-treatment hemoglobin, chemotherapy, and pelvic lymph node metastasis. The C-index of the line chart was 0.699 (95% CI 0.652–0.746). The areas under the receiver operating characteristic curves for 3- and 5-years survival were 0.751 and 0.724. The nomogram was in good concordance with the actual survival rates. The independent prognostic factors for overall survival in elderly patients with cervical cancer after radiotherapy were: tumor size, pre-treatment hemoglobin, chemotherapy, and pelvic lymph node metastasis. The novel prognostic nomogram based on these factors showed good concordance with the actual survival rates and can be used to guide personalized clinical treatment.

## Introduction

Cervical cancer is a common malignant tumor in women, worldwide^1^. Cervical cancer deaths are expected to reach 474,000 per year by 2030, of which approximately 85% will occur in low- and middle-income countries^2^. Numerous epidemiologic data have identified age as a clear risk factor for invasive disease, with spikes in incidence for women in their third and sixth decades of life^3^.

The World Health Organization defines elderly as aged 65 years or older. Elderly women account for about 25% of cervical cancer cases worldwide, and the 5-years survival rate is about 40.8%^4,5^. Diver et al.^6^ showed that age ≥ 65 years was an independent prognostic factor of cervical cancer. As the population ages, the number of elderly women with cervical cancer is also expected to increase, so therapeutic efficacy deserves further attention.

The physical status of elderly women with cervical cancer is exacerbated by other age-related conditions such as hypertension, diabetes, and heart disease^7^. According to the International Federation of Obstetrics and Gynecology and Obstetrics Staging Systems, radiotherapy for early cervical cancer is as effective as surgery, and radical radiotherapy and chemotherapy may be considered for advanced cases^8^. George et al.^9^ showed that rates of perioperative complications and mortality were higher in the older patients with cervical cancer than the younger. Advances in radiotherapy technology, specifically intensity-modulated radiation therapy (IMRT), has allowed a targeted dose to the tumor volume and less exposure of normal tissue^10^. Many studies have shown that radiotherapy is well tolerated in elderly patients with cervical cancer, even those aged 80 years or more^11^.

Overall, the prognosis of cervical cancer depends upon factors such as tumor size, clinical stage, pathological type, lymph node metastasis, depth of invasion, and others^12^. However, prognostic factors and the efficacy of treatment of cervical cancer in the elderly in China has received limited notice. Wang et al.^13^ reported that the clinical prognosis of elderly patients with cervical cancer degrades with decline of body function and autoimmune weakening, but the specific factors affecting prognosis are unknown. To provide clinicians with therapeutic guidance, it is important to explore these prognostic factors.

This retrospective review of 367 cases of elderly women with stage II–III cervical cancer after radiotherapy identified prognostic factors that were used to develop a prognostic model of 3- and 5-years survival.

## Methods

### Patients

**Figure 1:**
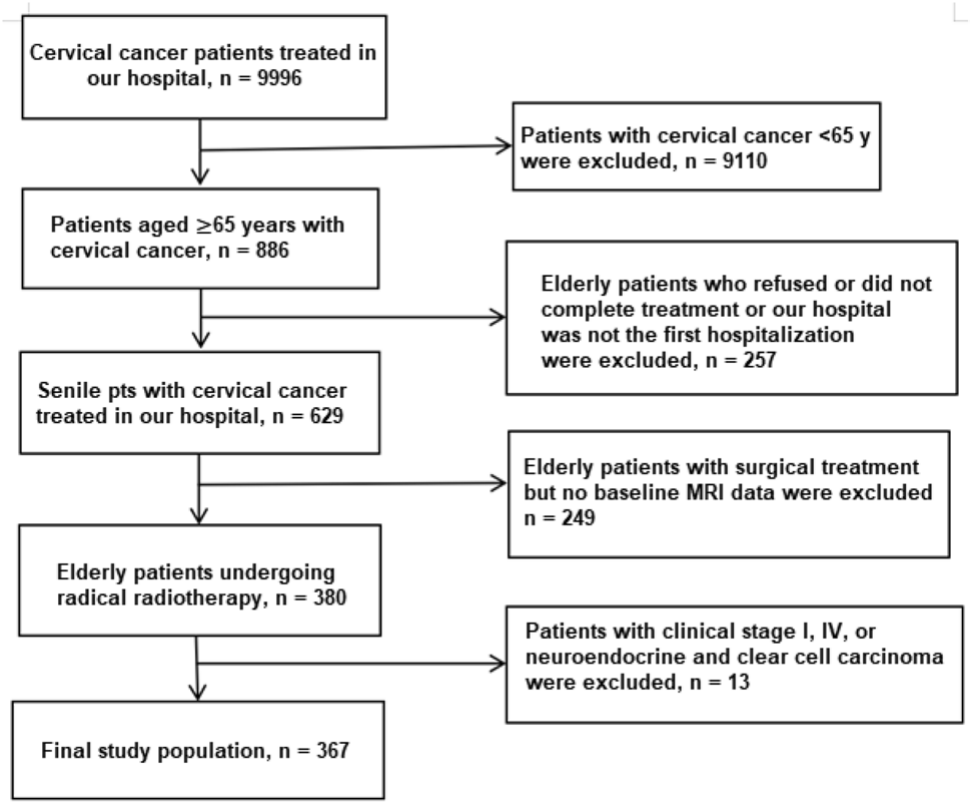
Schematic of the subject selection.

The total data of 9996 patients with cervical cancer who had been treated at our hospital between January 2012 and December 2016 were collected (Fig. 1). For the current study the selected subjects conformed to the following inclusion criteria: aged 65 years or older; with primary cervical cancer stage II–III (squamous cell carcinoma or adenocarcinoma); required and received radiotherapy; and with available pre-radiotherapy magnetic resonance imaging data. In addition, patients who underwent surgery without adequate radiotherapy, or with a history of other malignancy or pathological type, were excluded. Among the initial 9996 cases, 367 were eventually included and analyzed in this study. Informed consent was obtained from all subjects and/or their legal guardian. Additionally, this study was approved by the medical ethical committee review board of the Fujian Cancer Hospital (No. K2023-210-01). We confirmed that all methods were performed in accordance with the relevant guidelines and regulations.

### Treatment strategy

All the subjects were treated with radiotherapy and platinum-based chemotherapy. Radiotherapy consisted of either IMRT or conventional (4-field box conformal) radiotherapy. External whole-pelvis irradiation was performed with a dose of 1.8–2.0 Gy per fraction, 5 times per week, up to a total external dose of 45.0–50.0 Gy. After this, a high-dose-rate intracavitary bratherapy was administered at a weekly fractional dose of 7.0 Gy to a total dose of 28.0 Gy in 4 weeks. Chemotherapy was applied during radiotherapy, using cisplatin 60–75 mg/m^2^ every 3 weeks, or cisplatin in combination with paclitaxel 135 mg/m^2^ for 4 cycles.

### Data extraction and assessment

The data for patients’ characteristics and outcome-related factors were extracted and assessed. The primary endpoint of this study was overall survival (OS), considered the time from diagnosis of cervical cancer to death or the last follow-up. The secondary endpoints were local recurrence-free survival (LRFS) and distant metastasis-free survival (DMFS), defined the interval between the diagnosis of cervical cancer and, respectively, local recurrence or occurrence of distant metastasis.

### Nomograph model construction

Potential prognostic factors were analyzed using SPSS 26.0, and a nomogram survival spectrum was constructed and verified using R-3.6.3. The prognostic factors that were found significant (*P* < 0.05) as screened by the univariate Cox regression were included in the multivariate Cox regression analysis, upon which the nomogram model was constructed. The consistency index (C index) was calculated and the nomogram prediction model was tested. The higher the C index, the greater the AUC (area under the receiver operating characteristic curve, ROC) value.

### Statistical method

The changes in each parameter (independent and dependent groups) were compared using the t-test. The Kaplan–Meier method was employed for the LRFS, DMFS and OS analyses, and the survival curves were plotted using GraphPad Prism 8. COX regression was applied for univariate and multivariate analyses of prognosis and to estimate the hazard ratio (HR) and corresponding confidence interval (CI). *P* values < 0.05 were considered statistically significant. All statistical analyses were performed using SPSS 26.0 (SPSS, Chicago, Illinois).

### Ethics approval and consent to participate

This study was approved by the medical ethical committee review board of the Fujian Cancer Hospital (No. K2023-210-01).

## Results

### Characteristics of patients

The 9996 potential cases were screened according to the inclusion and exclusion criteria (Fig. 1). Staging was conducted according to the International Federation of Gynecology and Obstetrics (FIGO) staging standards of 2009 and 2018 (Table 1). The final population of 367 patients had a median age of 69 years (range 65–88 years).

**Table 1:** FIGO editions 2009 and 2018.

	FIGO 2009	FIGO 2018
IIa	37	35
IIb	152	138
IIIa	35	30
IIIb	143	105
IIIc1r	Nil	52
IIIc2r	Nil	7

The following factors were analyzed: chemotherapy; anemia status; SCC-Ag (before treatment); tumor size; pathological type; radiotherapy method; hypoprotein-emia; and infections and complications. Specifically, chemotherapy regimen was either with cisplatin alone or with cisplatin combined with paclitaxel. Anemia was defined as mild, moderate, or severe anemia. Tumor size was categorized as < 2, 2–4, or > 4 cm. Pathological types comprised squamous carcinoma, non-squamous carcinoma, or pelvic or retroperitoneal lymph node metastasis. Radiotherapy was either conformal or IMRT. Hypoproteinemia was defined as plasma albumin < 40.0 g/L. Infections and complications included hypertension, cardiovascular disease, and diabetes (Table 2).

**Table 2:** Clinical features of the patients.

	n (%)
FIGO 2018 clinical stage	
IIa	35 (9.54)
IIb	138 (37.60)
IIIa	30 (8.17)
IIIb	105 (28.61)
IIIc1r	52 (14.17)
IIIc2r	7 (1.91)
Age, years	
< 72	252 (68.66)
≥ 72	115 (31.34)
Chemotherapy	
Yes	160 (43.60)
No	207 (56.40)
Infection	
Yes	34 (9.26)
No	333 (90.74)
HB, g/L	
≥ 110	316 (86.10)
90 ≤ HB < 110	45 (12.26)
30 ≤ HB < 90	6 (1.64)
SCC before treatment, µg/L	
≤ 11.4	250 (68.12)
> 11.4	117 (31.88)
Tumor size, cm	
< 2	16 (4.36)
2–4	152 (41.42)
> 4	199 (54.22)
Pathologic type	
SCC	356 (97.00)
Not SCC^a^	11 (3.00)
Pelvic lymph node metastasis	
Yes	57 (15.53)
No	310 (84.47)
Retroperitoneal lymph node metastasis	
Yes	7 (1.91)
No	360 (98.09)
Radiotherapy mode	
CRT	225 (61.31)
IMRT	142 (38.69)
Hypoalbuminemia	
Yes	247 (67.30)
No	120 (32.70)
Complications^b^	
Yes	176 (47.96)
No	191 (52.04)

*HB* hemoglobin, *SCC* squamous cell carcinoma.

^a^Adenocarcinoma, adenosquamous carcinoma.

^b^e.g., hypertension, cardiovascular disease, diabetes.

### Survival outcome analysis

**Figure 2:**
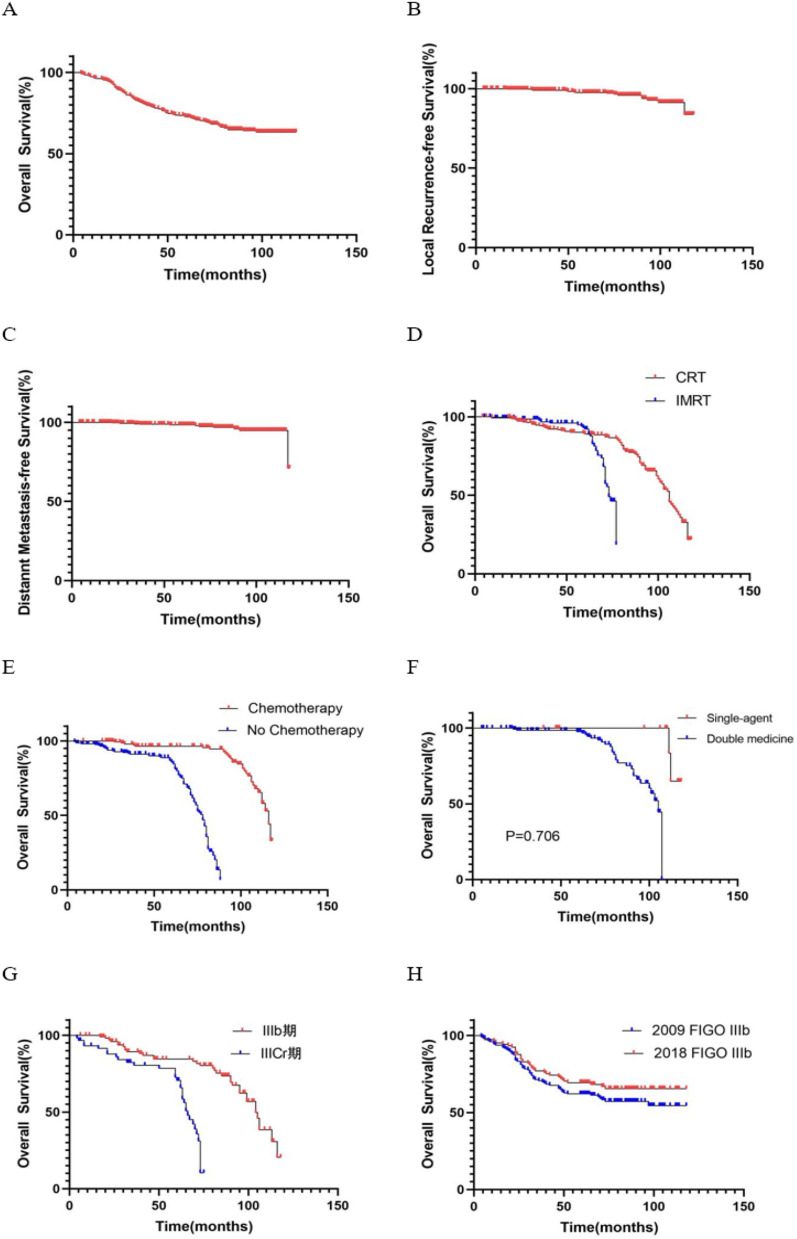
Survival curves. *Note*. (**A**) OS; (**B**) LRFS; (**C**) DMFS; (**D**) conformal radiotherapy (CRT) and IMRT; (**E**) chemo and no chemo; (**F**) single-agent and double-medicine chemotherapy; (**G**) OS for IIIb and IIIcr (FIGO 2018); (**H**) OS for IIIb (FIGO 2009 and FIGO 2018).

By December 2021, the median follow-up was 71 months (4–118 months). Of the 367 patients, 15 (4%) had local recurrence and 11 (3%) had distant metastasis The 3- (5-) years rates of OS, PFS, LRFS and DMFS were, respectively 91.0% (84.4%), 92.3% (85.9%), 99.18% (97.01%), and 99.18% (97.82%) (Fig. 2A–C). There was no significant difference in the choice of radiotherapy mode, whether it was conventional or IMRT (*P* = 0.173) (Fig. 2D). Concurrent chemo-radiotherapy significantly improved survival compared with radiotherapy alone (*P* < 0.001) (Fig. 2E), but no significant improvement was obtained between dual or single chemotherapy (*P* = 0.706) (Fig. 2F). FIGO staging 2018 showed that the 5-years OS of stage IIIb and IIIcr were 85% and 80%, respectively) (Fig. 2G). Compared with the FIGO stage definition of stage IIIb published in 2009, the revised definition of 2018 resulted in an increase in 5-years survival rate of 7.5%, from 62.5 to 70%) (Fig. 2H).

### Univariate prognostic analysis

The COX univariate study showed that the following were associated with OS: clinical stage, tumor size, age, hemoglobin, SCC-Ag, chemotherapy, and pelvic or retroperitoneal lymph node metastasis (all, *P* < 0.1; Table 3). The following showed no significant effect on prognosis: pathological type, radiotherapy mode, infection, hypoproteinemia, or related complications (*P* > 0.1).

**Table 3:** Univariate prognostic analysis affecting OS.

	HR (95% CI)	*P*
Tumor size, cm		
< 2	1 (ref)	
2–4	4.602 (0.633–33.476)	0.132
> 4	8.460 (1.177–60.795)	0.034
Hemoglobin value, g/L		
≥ 110	1 (ref)	
90 ≤ HB < 110	1.486 (0.909–2.429)	0.115
30 ≤ HB < 90	5.284 (2.143–13.027)	< 0.001
Age, years	1.496 (1.039–2.155)	0.030
Clinical staging	2.219 (1.520–3.240)	< 0.001
Pathologic type	1.618 (0.661–3.963)	0.292
Chemotherapy	0.373 (0.248–0.561)	< 0.001
Pelvic lymph node metastasis	2.434 (1.625–3.646)	< 0.001
Retroperitoneal metastasis	3.095 (1.361–7.036)	0.007
Co-infection	1.420 (0.813–2.478)	0.217
SCC-Ag, µg/L	1.797 (1.251–2.581)	0.002
Radiotherapy modality*	0.776 (0.532–1.133)	0.189
Hypoalbuminemia	1.187 (0.807–1.747)	0.384
Complications	0.865 (0.605–1.237)	0.426

*HB* hemoglobin.

*Conformal or IMRT.

### Multivariate prognostic analysis

All variables with *P* < 0.1 in the univariate analysis were included in the Cox multivariate analysis (Table 4). The following factors were significantly associated with prognosis: tumor size, hemoglobin, chemotherapy, and pelvic lymph node metastasis. Tumor size > 4 cm (*P* = 0.031), and moderate and severe anemia (*P* < 0.001) suggest a significant correlation between prognosis. Chemotherapy and pelvic lymph node metastasis were significantly associated with prognosis (*P* < 0.001).

**Table 4:** Results of multivariate prognostic analysis affecting OS.

	HR (95% CI)	*P*
Tumor size, cm		
< 2	1 (ref)	–
2–4	7.008 (0.937–52.414)	0.058
> 4	9.082 (1.226–67.305)	0.031
Hemoglobin value, g/L		
≥ 110	1 (ref)	–
90 ≤ HB < 110	1.131 (0.670–1.908)	0.582
30 ≤ HB < 90	5.529 (2.012–15.193)	< 0.001
Chemotherapy*	0.328 (0.211–0.510)	< 0.001
Age, years	1.127 (0.749–1.696)	0.565
Pelvic lymph node metastasis	2.275 (1.410–3.672)	0.001
Clinical staging	1.283 (0.805–2.047)	0.295
Retroperitoneal lymph node metastasis*	1.621 (0.665–3.951)	0.288
SCC-Ag	1.255 (0.842–1.870)	0.264

SPSS Spearman method was used to analyze the correlation between clinical stage, tumor size and lymph node metastasis, and the correlation coefficients were (r = 0.412, r = 0.198). The results showed that clinical stage and tumor size were correlated with lymph node metastasis (*P* < 0.001).

*HB* hemoglobin.

*Yes or no.

### Analysis of related factors for lymph node metastasis

The Spearman analysis showed associations between lymph node metastasis and clinical stage (r = 0.412) and tumor size (r = 0.198; both *P* < 0.001).

### Construction and verification of the nomograph

**Figure 3:**
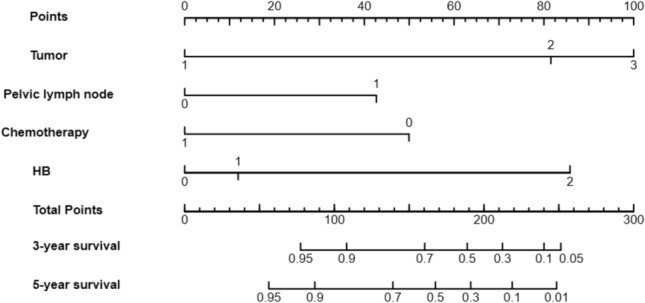
The line chart mode. *Note*. In HB, 0 represents HB ≥ 110 g/L, 1 represents HB: 90 g/L ≤ HB < 110 g/L, 2 represents HB: 30 g/L ≤ HB < 90 g/L; In Tumor, 1 represents the mass size < 2 cm, 2 represents the mass size between 2 and 4 cm, and 3 represents the mass size > 4 cm. For researcher node, 0 means no transfer and 1 means transfer. In Chemotherapy, 0 means no Chemotherapy, 1 means Chemotherapy.

**Figure 4:**
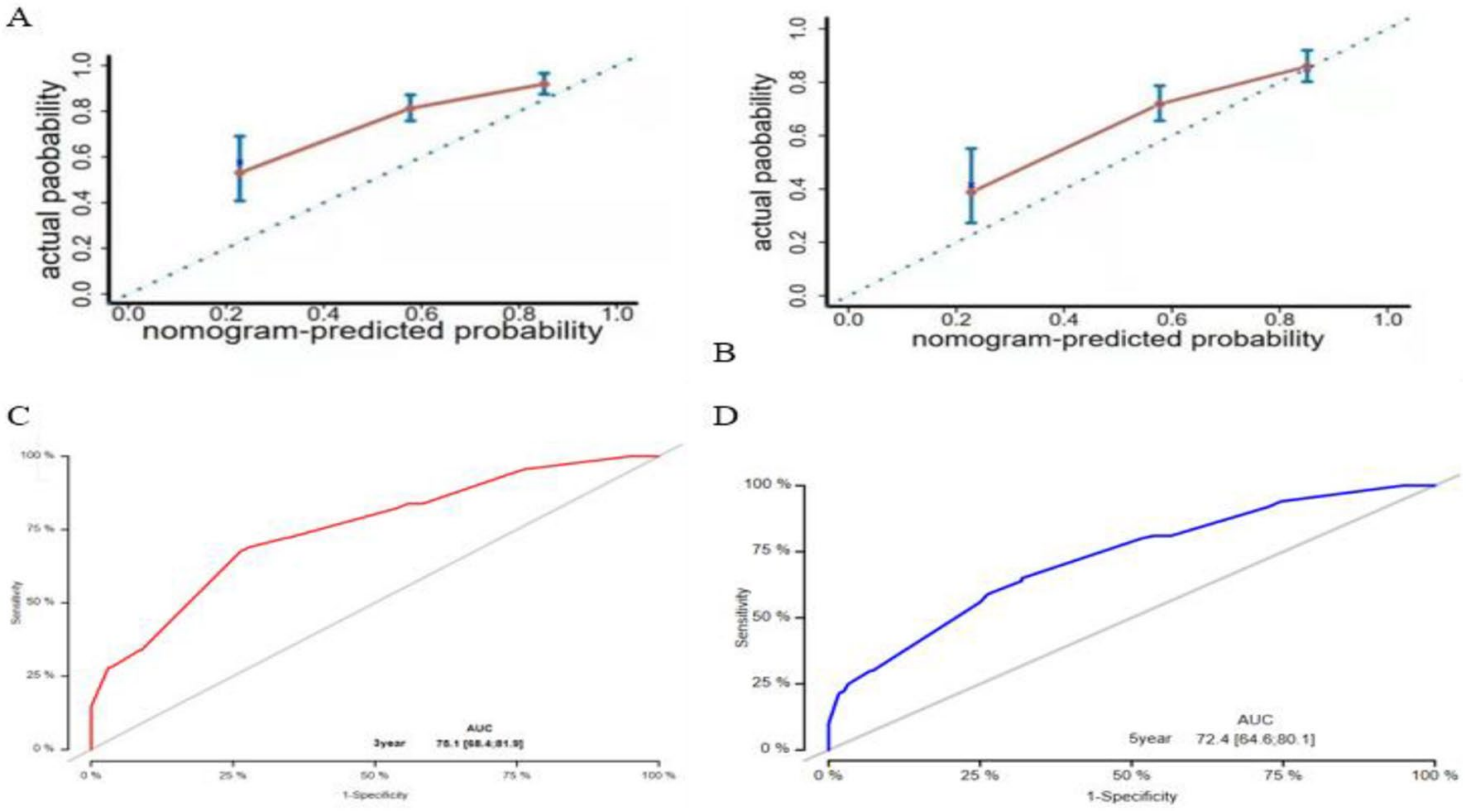
Curves of the predictive model. *Note*. (**A**) Calibration, 3-years. (**B**) Calibration, 5-years. (C) ROC, 3-years. (**D**) ROC, 5-years.

A prognostic nomogram that integrated all the independent predictors for OS was constructed (Fig. 3). The C-index was 0.699 (95% CI 0.652–0.746). The 3-, and 5-years AUC values of the nomogram were respectively 0.751 and 0.724 (Fig. 4). Patient inclusion and exclusion process, suggesting high consistency between the predictive ability of the nomogram and the actual survival rates.

## Discussion

The incidence and mortality rate of cervical cancer ranks fourth in the world among the malignant tumors of women in the world^1^. With the emergence of aging society, the new cases of cervical cancer in the elderly are gradually increasing^14^. Therefore, it is of great significance to explore the risk factors and appropriate treatment for the elderly cervical cancer.

The prognosis of cervical cancer is multifactorial. Yang et al.^15^ concluded that the independent prognostic factors in stage IB-IIA cervical cancer under postoperative were number of complications, surgical methods, neoadjuvant treatment, lymph node metastasis, lymphovascular space invasion (LVSI). In our study, the five factors affecting the prognosis of the elderly cervical cancer received radiotherapy were tumor size, age, HB value, chemotherapy, and pelvic lymph node metastasis. It shows that under the premise of controlling side effects, elderly cervical patients should be encouraged to undergo concurrent chemoradiotherapy, especially for the patients with big tumor size, pelvic lymph node metastasis and late stage. The univariate analysis showed that clinical stage was significantly associated with prognosis (*P* < 0.001). However, the Cox multivariate analysis of the present study showed no such association. This may be due to selection bias related to the small sample size of a retrospective analysis, or the advanced stage of these elderly patients.

Radiotherapy is the best choice for the elderly with cervical cancer because of their poor physical condition and comorbidities such as hypertension, diabetes, and heart disease. Sharma et al.^16^ also reported the advantages of radiotherapy in the elderly with cervical cancer. In our study, the survival of patients received IMRT was similar to that of those with conventional radiotherapy (*P* = 0.189).

Lin et al.^17^ reported that the rates of adverse reactions such as radiation cystitis, colitis, and bone marrow suppression in the IMRT group were significantly lower compared with that of the group given conventional radiotherapy. Shi et al.^18^ also confirmed that the occurrence of grade 2 gastrointestinal reactions was reduced from 91 to 60%, and grade 3 gastrointestinal toxicity was significantly reduced from 50 to 11.1% compared with ventional radiotherapy (*P* = 0.189).

A meta-analysis by Datta et al.^19^ showed that the 5-years overall survival rate of concurrent chemo-radiotherapy in locally advanced cervical cancer increased by 7.5% compared with radiotherapy alone. In our study, the 5-years survival of concurrent chemo-radiotherapy was improved by 20% (85% and 65%) than that of radiotherapy alone in elderly patients (*P* < 0.05). However, in terms of drugs, there was no significant difference in prognosis between patients given chemotherapy with dual agents or single agents (*P* = 0.706).

Liu et al.^20^ reported that anemia before radiotherapy were predictors of the OS (*P* = 0.008). Anemia can lead to ischemia at the site of the malignant tumor, thus reducing the local control rate of radiotherapy and chemotherapy. At the same time, hypoxia changes the local protein and genome structure of malignant tumor sites, thus improving the invasion ability of malignant tumor cells^21^. High hemoglobin content can improve the oxygen supply and promote the transformation of hypoxic cells into proliferative cells, thus improving the efficacy of radiotherapy and chemotherapy in malignant tumors. In our study, 51 cases (13.86%) of moderate and severe anemia had significant influence on survival and prognosis (*P* < 0.001). So in middle-aged and elderly patients with radiotherapy, anemia should be improved as far as possible to achieve better radiotherapy efficacy.

In pre-cervical cancer studies, SCC-Ag could effect the survival and prognosis of cervical squamous cell carcinoma. Chen et al.^22^ showed that the 5-years OS and PFS of patients with SCC_pre_ < 11.4 g/L and SCC_post_ < 1.9 g/L were better than the group which SCC_pre_ > 11.4 g/L and SCC_post_ > 1.9 g/L. The univariate analysis showed that SCC_pre_ < 11.4 μg/L was a significant prognostic indicator.

Our study showed that the correlation coefficient between tumor size and lymph node metastasis was 0.198 (*P* < 0.001). This result agrees with Cai et al.’ study^23^ and clinical stage also was significantly associated with lymph node metastasis (r = 0.412, *P* < 0.001).

Our study developed a nomogram to estimate the probability of OS for patients with staged II-III cervical cancer who received radiotherapy with or without adjuvant chemotherapy. The nomogram showed high prognostic efficacy and good reproducibility. As the score increased, OS significantly worsened. The nomogram is based on multifactorial regression analysis. The complex regression equation is transformed into visual images to make the conclusions of the prediction model more readable and convenient for doctors to evaluate patients^24,25^. In the present study, univariate and multivariate Cox regression analyses were used to establish five independent prognostic indicators (tumor size, age, chemotherapy, anemia, and pelvic lymph node metastasis), which were incorporated into a Nomogram prediction model. To evaluate the degree of differentiation of prediction models, C-index statistics and ROC curve drawing are usually used to compare and verify models^26^. In our study, the C-index of the model is 0.699 and the average AUC values for 3- and 5-years OS are 0.751 and 0.724, respectively, which indicating that the prediction model has high resolution. The calibration curve also showed that the predicted 3-years and 5-years survival rates were very close to the actual patient survival rates, suggesting that the prediction model is acceptably accurate.

The limitations of the present study are that it is a retrospective analysis based on data from a single center with a small sample. Therefore, the selection of relevant variables may be biased. Furthermore, there is no external test data to verify the nomograph. For validation, further studies are warranted to improve accuracy and popularize the model.

## Conclusions

Clinical stage, tumor size, hemoglobin value, chemotherapy and pelvic lymph node metastasis were independent factors affecting the prognosis of elderly patients with cervical cancer. The prognostic nomogram showed good concordance with the actual survival rates and may help guide clinical treatment.

## Data Availability

The data used or analyzed during the current study are available from the corresponding author on reasonable request.

## Abbreviations

AUC: Area under the receiver operating characteristic curve
CI: Confidence interval
C-index: Consistency index
DMFS: Distant metastasis-free survival
FIGO: International Federation of Gynecology and Obstetrics
HR: Hazard ratio
IMRT: Intensity-modulated radiotherapy
LRFS: Local recurrence-free survival
OS: Overall survival
ROC: Receiver operating characteristic
SCC-Ag: Squamous cell carcinoma antigen
